# CoViD vaccines and thrombotic events: EMA issued warning to patients and healthcare professionals

**DOI:** 10.1186/s40545-021-00315-w

**Published:** 2021-03-24

**Authors:** Hamid A. Merchant

**Affiliations:** Department of Pharmacy, School of Applied Sciences, University of Huddersfield, Queensgate, Huddersfield, HD1 3DH United Kingdom

There have been recent reports of haemorrhage, blood clots and thrombocytopenia following administration of CoViD-19 vaccines that have raised concerns over the safety of genetic vaccines for people with pre-existing coagulation disorders or those on certain medications. This had also led to temporary suspension of Oxford/AZ CoViD vaccine in a number of countries across Europe [1]. As a result, Medicine and Healthcare Regulatory Authority, UK (MHRA) and European Medicines Agency (EMA) initiated a rigorous scientific review of pharmacovigilance data and available evidence to assess the potential association of the vaccine to recent thrombotic events.

MHRA confirmed on 18th March 2021 that the ‘evidence does not suggest that blood clots are caused by COVID-19 Vaccine AstraZeneca and assured that the benefits of the vaccine in preventing COVID-19 far outweigh the thrombotic risks and people should continue taking the vaccine when offered [2].

Medicine and Healthcare Regulatory Authority (MHRA) further stated that sinus vein thrombosis along with thrombocytopenia has been reported in less than one in a million people vaccinated so far in the UK, it can occur naturally and that a causal association with the vaccine could not be established. However, they will continue to monitor the situation, and as a precautionary measure advised that anyone with a headache lasting more than 4 days after vaccination, or bruising beyond the site of vaccination, should seek medical attention.

EMA on the other hand also investigated the events and concluded on 18th March 2021 that the ‘vaccine may be associated with very rare cases of blood clots associated with thrombocytopenia with or without bleeding, including rare cases of clots in the vessels draining blood from the brain’ [3]. EMA stated that a causal link with the vaccine is ‘not proven but possible’ and deserves further analysis. They, however, also reassured that the benefits of having a CoViD vaccine still outweigh the risks despite a possible link to the rare blood clots with low blood platelets. They also assured a continued monitoring of the situation and further review of thrombotic risk with other CoViD vaccines.

EMA has also taken additional measures to include information on thrombotic risks in the vaccine’s summary of product characteristics (SmPC) and product information leaflets. Furthermore, EMA issued warnings to the patients and healthcare professionals to be vigilant. EMA also published an extended list of symptoms which, if found persistent beyond 3 days of vaccination, patients should seek a prompt medical assistance. The list of symptoms included breathlessness, pain in the chest or stomach, swelling or coldness in an arm or leg, severe or worsening headache or blurred vision, persistent bleeding, multiple small bruises, reddish or purplish spots, or blood blisters under the skin. EMA urged the healthcare professionals to be alert for potential risk of thromboembolism in vaccinated individuals.

There are reports of antibodies present in CoViD-19 patients that activated platelets [4] and patients with thrombocytopenia following CoViD vaccination showed a favourable response to immune thrombocytopenia-directed therapies (corticosteroids and IVIG) [5]. We proposed a likely mechanism on 15th March 2020 that the genetic CoViD-19 vaccines may directly infect platelets and megakaryocytes triggering mRNA translation and consequent spike protein synthesis intracellularly. This may potentially result in an autoimmune response against platelets and megakaryocytes resulting in reticuloendothelial phagocytosis and direct CD8+ T cell lysis [6]. The consequent thrombocytopaenia may lead to internal bleeding and spontaneous blood clots. On 19th March 2020, scientists from Oslo identified an antibody from vaccinated individuals which they suspect being responsible for attacking platelets and causing recent thrombotic events [7]. This discovery also supports our hypothesis [6] that CoViD genetic vaccines may have a direct role in spurring autoimmune response against platelets that may clinically manifest in thrombocytopenia, haemorrhage, and blood clots.

Vaccines are one of the great discoveries in medicine that has improved life expectancy dramatically. Nonetheless, genetic vaccines are new, and their long-term safety evaluation is a key to identify potentially contraindicated group of subjects, for instance, patients with history of blood disorders, past or current thrombocytopenia or pre-existing immunological conditions.

Pharmacovigilance can play a key role in early detection of potentially severe adverse events. The recent experience with Pandemrix raised fundamental questions on the regulatory approach to pharmacovigilance. It reminds us of the duty of the public health agencies to warn the public over possible harms of vaccines, the level of details that public should be made aware of, who should provide the information to public, and whether the provision of such information be proactive or passive [8]. The early signs of rare side effects during pharmacovigilance that may lead to severe adverse outcomes should never be dismissed just on the basis of prevalence statistics, but require thorough scientific investigations and clinical correlation to rule out a potential causal link. We, therefore, welcome EMA’s stance on issuing a caution to the public and physicians and support their decision of continued monitoring of thrombotic events more closely with necessary scientific investigations.
